# Error in Byline

**DOI:** 10.1001/jamanetworkopen.2021.20854

**Published:** 2021-07-06

In the Invited Commentary titled “Addressing Social Risk and Support as Adjuvants in Colorectal Cancer Treatment,”^1^ published June 9, 2021, the first author’s name should have appeared as Fritz Francois, not Fritz François. This article has been corrected.^1^
